# Evaluating the Performance of Hyperspectral Imaging Endoscopes: Mitigating Parameters Affecting Spectral Accuracy

**DOI:** 10.3390/bios15110738

**Published:** 2025-11-04

**Authors:** Siavash Mazdeyasna, Mohammed Shahriar Arefin, Andrew Fales, Silas J. Leavesley, T. Joshua Pfefer, Quanzeng Wang

**Affiliations:** 1 Center for Devices and Radiological Health, U.S. Food and Drug Administration, Silver Spring, MD 20993, USA; siavash.mazdeyasna@gmail.com (S.M.); mohammedshahriar.arefin@fda.hhs.gov (M.S.A.); andrew.fales@fda.hhs.gov (A.F.); joshua.pfefer@fda.hhs.gov (T.J.P.); 2 Chemical and Biomolecular Engineering, University of South Alabama, Mobile, AL 36688, USA; leavesley@southalabama.edu

**Keywords:** medical hyperspectral imaging, hyperspectral endoscope, reflectance spectrum, interferometer-based spectral scanning, snapshot, spectral accuracy, spectral noise, optimization, normalization, ambient light, exposure time, camera warm-up time, spatial and temporal averaging, camera focus, working distance, illumination angle, target angle

## Abstract

Hyperspectral imaging (HSI) is increasingly used in studies for medical applications as it provides both structural and functional information of biological tissue, enhancing diagnostic accuracy and clinical decision-making. Recently, HSI cameras (HSICs) have been integrated with medical endoscopes (HSIEs), capturing hypercube data beyond conventional white light imaging endoscopes. However, there are currently no cleared or approved HSIEs by the U.S. Food and Drug Administration (FDA). HSI accuracy depends on technologies and experimental parameters, which must be assessed for reliability. Importantly, the reflectance spectrum of a target can vary across different cameras and under different environmental or operational conditions. Thus, before reliable clinical translation can be achieved, a fundamental question must be addressed: can the same target yield consistent spectral measurements across different HSI systems and under varying acquisition conditions? This study investigates the impact of eight parameters—ambient light, exposure time, camera warm-up time, spatial and temporal averaging, camera focus, working distance, illumination angle, and target angle—on spectral measurements using two HSI techniques: interferometer-based spectral scanning and snapshot. Controlled experiments were conducted to evaluate how each parameter affects spectral accuracy and whether normalization can mitigate these effects. Our findings reveal that several parameters significantly influence spectral measurements, with some having a more pronounced impact. While normalization reduced variations for most parameters, it was less effective at mitigating errors caused by ambient light and camera warm-up time. Additionally, normalization did not eliminate spectral noise resulting from low exposure time, small region of interest, or a spectrally non-uniform light source. From these results, we propose practical considerations for optimizing HSI system performance. Implementing these measures can minimize variations in reflectance spectra of identical targets captured by different cameras and under diverse conditions, thereby supporting the reliable translation of HSI techniques to clinical applications.

## 1. Introduction

Endoscopes are essential for minimally invasive procedures, enhancing patient safety [1] and aiding disease diagnosis and treatment [2,3]. Conventional white light endoscopes provide only spatial information about biological tissues and lack spectral detail, limiting tissue differentiation and diagnostic accuracy. To address this limitation, image-enhancement techniques such as narrow band imaging [4,5] and autofluorescence imaging [6] have been developed to improve visualization and diagnostic capability. However, these methods rely on a limited set of predefined spectral bands and cannot capture full tissue spectra.

Recently, hyperspectral imaging (HSI) has been applied across various commercial and scientific fields, including quality control in the food industry, satellite imaging, and environmental monitoring [7,8], as HSI provides valuable spectral and spatial information simultaneously. HSI systems employ various techniques, including spatial scanning, spectral scanning and snapshot imaging, to acquire spatial–spectral data, commonly referred to as hypercubes [9]. Spatial-scanning techniques include whiskbroom (point-scanning) and pushbroom (line-scanning) approaches. The whiskbroom approach captures spectral data point-by-point by scanning across two spatial dimensions (x and y), while the pushbroom approach acquires spectral data line-by-line by scanning in a single spatial direction to generate a full hypercube. These approaches achieve wavelength separation using dispersive elements such as prisms, diffraction gratings, or a combination of both, like a prism-grating-prism (PGP) configuration. Spectral-scanning techniques, in contrast, capture spatial data in each frame while sequentially scanning across a range of wavelengths to compile a hypercube. These techniques typically employ tunable filters or interferometers, such as acousto-optic tunable filters (AOTF), liquid crystal tunable filters (LCTF), or Fabry–Perot interferometers (FPI), allowing precise selection of narrow spectral bands. Detailed explanations of different tunable filters and interferometers are provided in references [10,11]. Unlike spatial- or spectral-scanning techniques, snapshot imaging captures the entire hypercube in a single acquisition without the need for scanning. The advantages and disadvantages of these HSI techniques have been discussed in detail by others [9,12,13].

The ability of HSI to simultaneously capture spatial and spectral information has made it invaluable for medical applications. In healthcare, HSI has demonstrated potential in cancer detection by distinguishing tumor tissues from healthy ones based on their unique spectral signatures [14]. HSI has also been used in retinal imaging for disease detection [15], tissue oxygenation assessment for wound care [16], and surgical guidance [17]. In endoscopy, HSI can play a crucial role in differentiating tissue types, aiding in the detection of abnormalities that may not be visible with conventional imaging techniques [14,18,19]. In spite of this strong potential for improving patient outcomes, practical challenges remain.

Reliable performance in HSI endoscopy requires high-fidelity hypercubes, yet these can be influenced by illumination factors (e.g., light source spectrum, illumination angle, ambient light) [20,21,22,23,24,25], camera settings (e.g., warm-up, exposure time, focus, working distance, imaging angle) [9,22,26,27,28,29], and data analysis methods [25]. Variations in these parameters may destabilize signals, introduce artifacts, and distort spectra, thus compromising diagnostic performance. Because endoscopic applications demand precise and reproducible performance, controlled bench studies are needed to evaluate these effects and develop mitigation methods. While prior work has examined individual parameters [9,23,24,25,28,29], systematic strategies to mitigate their impact in medical HSI remain largely unexplored.

The objective of this study was to develop a set of test methods to evaluate the effects of eight key parameters—ambient light, exposure time, camera warm-up time, spatial and temporal averaging, camera focus, working distance (WD), illumination angle, and target angle—on the spectral accuracy of HSI systems. Controlled experiments were conducted using two HSI techniques, interferometer-based spectral scanning and snapshot imaging, in two phases, with Phase 1 focusing on two HSI cameras (HSICs) and Phase 2 focusing on two customized experimental HSI endoscopes (HSIEs). We also investigated whether normalization could mitigate the effects of these parameters. The goal of this work was to reduce discrepancies in reflectance spectra of the same target acquired with different systems (HSICs and HSIEs) and under varying conditions, thereby supporting more reliable application of HSI in clinical settings.

## 2. Methodology

We designed a series of experiments to evaluate and mitigate key parameters affecting the spectral accuracy of HSI systems, aiming to establish a reliable testing framework for optimization and robustness. The study included two phases and two HSI techniques: interferometer-based spectral scanning and snapshot imaging.

### 2.1. Experimental Setup and Design

The main equipment used in this study included two HSICs from two companies (Hinalea Imaging Corp., Emeryville, CA, USA; Cubert GmbH, Ulm, Germany); a rigid endoscope (Stryker Corp., Portage, MI, USA); an endoscope coupler (TTI Medical, San Ramon, CA, USA); a xenon light source (Smith & Nephew, Inc., London, UK); a tungsten-halogen (TH) light source (Thorlabs, Inc., Newton, NJ, USA); and several diffuse reflectance targets (Red, White, Erbium Oxide) with validated reflectance spectra (Labsphere, Inc., North Sutton, NH, USA). A complete list of equipment used in this study is provided in Table 1, while Table A1 in Appendix A summarizes further specifications of the two HSICs. HSICs can operate across different spectral ranges, from ultraviolet to near-infrared. We limited our analysis to the 450–830 nm range to ensure compatibility with the spectral ranges of both HSICs, the rigid endoscope and coupler, as well as the reference spectra. Spectral sampling was performed at each HSIC’s highest resolution: 2 nm for C-1 and 8 nm for C-2. For visualization and normalization of the spectra, the wavelength spacing of reference spectra was set to 2 nm.

**Figure 1:**
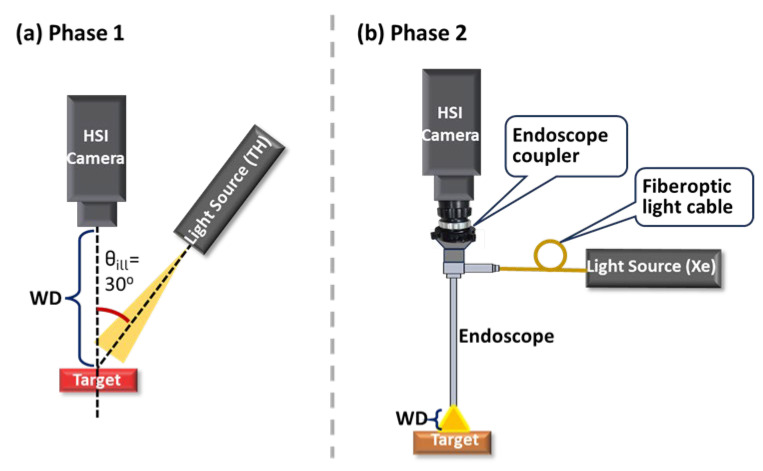
System setups in two study phases. (**a**) Phase 1: C-1 and C-2; (**b**) Phase 2: E-1 and E-2.

**Figure 2:**
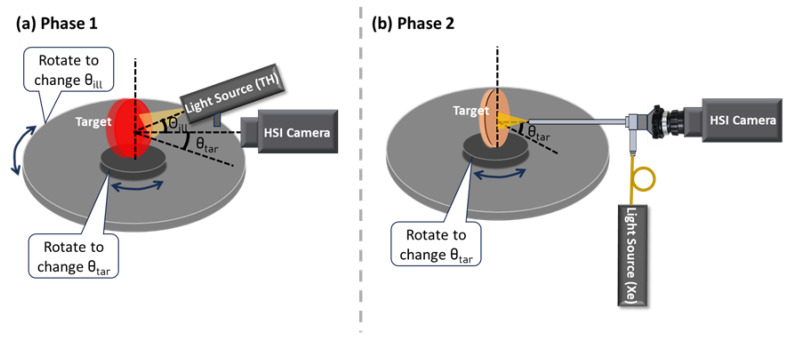
System setups in two study phases for evaluating $\theta_{ill}$ (angle between illumination beam and target surface normal) and $\theta_{tar}$ (angle between camera optical axis and target surface normal). (**a**) Phase 1: C-1 and C-2; (**b**) Phase 2: E-1 and E-2.

The study was performed in two phases. Phase 1 focused on the HSICs (C-1 and C-2) themselves (Figure 1 and Figure 2), with a default WD of 150 mm for C-1 and 90 mm for C-2. The Red target was used, with the default target angle ($\theta_{tar}$, the angle between the camera’s optical axis and the target surface normal) set to 0°. The TH light source was used in this phase, with the default illumination angle ($\theta_{ill}$, the angle between the illumination beam and the target surface normal) as 30°.

In Phase 2, the C-1 and C-2 cameras were integrated with the rigid endoscope via the endoscope coupler, forming two HSIEs (E-1 and E-2) for performance evaluation. The default WD for both E-1 and E-2 was 10 mm. To incorporate complex reflectance peaks found in biological tissues [30], we used the erbium oxide (EO) target in Phase 2. The target remained perpendicular to the HSIE optical axis ($\theta_{tar}$ = 0°). The Xe light source, delivered through a fiberoptic light cable and coupled to the illumination path of the rigid endoscope, was also aligned with the HSIE optical axis ($\theta_{ill}$ = 0°).

The HSI systems were positioned perpendicular to the table for all measurements (Figure 1), except when assessing $\theta_{ill}$ or $\theta_{tar}$ (Figure 2). Both the systems and the target platform were mounted on a vertically adjustable linear stage to control the WD. For $\theta_{ill}$ and $\theta_{tar}$ evaluations, the HSI systems were fixed horizontally to the table using a post. Two independently rotating stages, aligned along the same rotation axis, were used to control $\theta_{ill}$ and $\theta_{tar}$. The light source was mounted on the periphery of a larger rotating breadboard (RBB18A, Thorlabs, Inc., Newton, NJ, USA), allowing $\theta_{ill}$ adjustment. The target was vertically positioned on a smaller rotating platform (XYR1, Thorlabs, Inc., Newton, NJ, USA), ensuring that the axis of rotation passed through the target surface, enabling $\theta_{tar}$ adjustment. Unless noted, cameras and light sources were warmed up for one hour, and measurements were performed in a dark room. The exact exposure time and WD for each parameter studied in each phase are listed in Table A2 in Appendix A. The selection of exposure times aimed to maximize signal intensity while avoiding saturation in any spectral band.

### 2.2. Data Analysis Methods

HSI intensity data were converted to reflectance data using the following equation [13,31]:

(1) $$R(x,y,\lambda )=\frac{I(x,y,\lambda )-I_{dark}(x,y,\lambda )}{I_{w}(x,y,\lambda )-I_{dark}(x,y,\lambda )}$$

where R(x,y,λ) represents the reflectance at a specific spatial position (x,y) and wavelength (λ). I, $I_{w}$, and $I_{dark}$ denote the intensities of the target signal, the signal measured using a white reflectance target [13], and the dark signal recorded without illumination or with a blocked sensor, respectively. Since $I_{w}$ depends on experimental conditions, ideally, it should be remeasured whenever setting parameters or external conditions (e.g., exposure time, illumination spectrum and angle, WD) change. $I_{dark}$ is affected by camera temperature and exposure time [13] and therefore ideally should be remeasured when these parameters change.

To reduce temporal noise, each measurement was repeated three times under the same conditions, and the results were averaged to generate a hypercube. To minimize spatial signal-to-noise ratio (SNR) variation and outliers, we averaged 200 × 200 pixels for C-1/E-1 and 80 × 80 pixels for C-2/E-2 per spectral bands in the hypercube to compute signal intensity for reflectance calculations. As C-1 has a higher pixel resolution (1936 × 1216) than C-2 (275 × 290), we selected a larger region of interest (ROI, in pixel number) for C-1/E-1. In contrast, selecting a ROI of 200 × 200 pixels in C-2 would have extended beyond the diffuse reflectance target. Thus, we selected an ROI of 80 × 80 pixels for C-2/E-2. There was no established rule for ROI size selection; the exact pixel numbers do not affect the conclusion in this study. In clinical applications, the pixel number should be determined based on the specific scenario as summarized in the table provided in the Conclusions section. We further discuss the effect of spatial averaging in Section 3.4.

Our previous study [25] demonstrated that normalization is essential for HSI data analysis and cross-system comparisons. We evaluated nine normalization methods and found that the Standard Normal Variate (SNV) method slightly outperformed other eight methods in a benchtop setting. The SNV normalization equation is:

(2) $$R^{\prime }=\;\frac{R-\mu \left(R\right)}{\sigma \left(R\right)}$$

where μ(R) and σ(R) are the mean and standard deviation of the reflectance values, respectively. Following normalization, we introduced a uniform scaling step to standardize the range of R′ spectra while preserving relative spectral relationships and enhancing the robustness of spectral analysis. The R′ after uniform scaling is referred to as Scaled R′.

In this study, SNV normalization followed by the uniform scaling step was applied to convert R to Scaled R′, reducing amplitude variations from various parameters. This approach enabled cross-variation comparison and provided a framework to minimize spectral amplitude differences from the same target.

### 2.3. Parameters Affecting Spectral Accuracy

The influence of various system component and experimental parameters on the spectral performance of HSI systems was studied in two phases, focusing on HSICs and HSIEs, respectively. Table 2 lists the parameters examined in each phase. Repeatability and spatial/temporal averaging analyses were excluded from Phase 1 because the corresponding analyses in the more complex HSIEs in Phase 2 were more representative. Camera warm-up time was not studied in Phase 2, as this parameter pertains only to the camera and was already examined in Phase 1. The illumination angle was also not studied in Phase 2 because the endoscope’s light source angle is fixed and concentric with the illumination fiber. For HSICs, the Red target was used, whereas for HSIEs, the EO target was selected to introduce spectral complexities similar to those observed in biological tissues.

The exposure time and WD were carefully adjusted to ensure adequate signal intensity and measurement consistency. Their exact values are provided in Table A2 in Appendix A. The study for each parameter and each HSIC/HSIE was performed under two scenarios: (1) an ideal scenario, where $I_{w}$ and I in Equation (1) were measured under the same conditions, and (2) a realistic scenario, where $I_{w}$ was measured under an ideal condition, but I was measured under different conditions. The dark signal $I_{dark}$ in Equation (1) was always recorded with a blocked sensor. Original reflectance spectra (R) were calculated under both ideal and realistic scenarios, normalized with the SNV method (R′), and uniformly scaled to obtain scaled R′. R and scaled R′, collectively termed reflectance spectra, were compared with the reference spectra (Ref) traceable to the National Institute of Standards and Technology (NIST), as provided by the target manufacturers.

#### 2.3.1. Ambient Light

To evaluate the impact of ambient light on the spectral performance of the HSICs and HSIEs, $I_{w}$ and I were measured under two conditions: with and without ambient light (i.e., room light on or off). Reflectance in the ideal scenario was calculated when $I_{w}$ and I were measured under identical lighting conditions, either in complete darkness (Dark) or with ambient light (Amb). Reflectance in the realistic scenario was calculated when $I_{w}$ was measured in Dark, while I was measured with Amb.

#### 2.3.2. Exposure Time

Camera exposure (or integration) time exhibits a direct, often linear relationship with spectral intensity [22,27]. The optimal exposure time varies depending on the specific application and system’s capability [32]. In this study, we investigated the impact of exposure time on reflectance by measuring $I_{w}$, $I_{dark}$ and I with four different exposure times, as specified in Table A2. Reflectance in ideal scenarios was calculated when $I_{w}$, $I_{dark}$ and I were measured at the same exposure times. Reflectance in realistic scenarios was calculated when $I_{w}$ and $I_{dark}$ were measured at 500 ms for C-1, 100 ms for C-2, 500 ms for E-1, and 200 ms for E-2, while I was measured at a different exposure time. Different exposure times for $I_{w}$ measurements were selected to maximize output while avoiding saturation in any spectral band or gap.

#### 2.3.3. Camera Warm-Up Time

To investigate the impact of camera warm-up time, C-1 or C-2 was turned on, and intensity data were acquired at 10-min intervals from the White (for $I_{w}$ measurement) and Red (for I measurement) targets. Reflectance spectra were calculated under two scenarios: in ideal scenarios, $I_{w}$, $I_{dark}$ and I were measured at almost the same time at each warm-up interval, whereas in realistic scenarios, $I_{w}$ and $I_{dark}$ were measured at the beginning of the camera warm-up time period, while I was measured at different warm-up times. Camera sensor temperature was recorded throughout the warm-up period using the camera control software, as it can influence dark noise.

#### 2.3.4. Spatial and Temporal Averaging

Spatial and temporal averaging can improve SNR by reducing noise, thereby enhancing spectral accuracy. To analyze a hypercube, an ROI was selected from each spectral frame, and the pixel intensity values within the ROI were averaged to calculate $I_{w}$, $I_{dark}$ and I at each wavelength. To evaluate the impact of spatial averaging on noise levels in the reflectance spectra, the EO target data acquired with E-1 and E-2 were analyzed using two ROI sizes: the default size of 200 × 200 pixels for E-1 and 80 × 80 pixels for E-2, and a smaller size of 2 × 2 pixels for both E-1 and E-2. Throughout the study, a conservative approach was taken by calculating each reflectance spectrum based on the average of three measurements. To evaluate the effect of temporal averaging, the spectra derived from three measurements were compared with those obtained from a single measurement.

#### 2.3.5. Camera Focus

**Figure 3:**
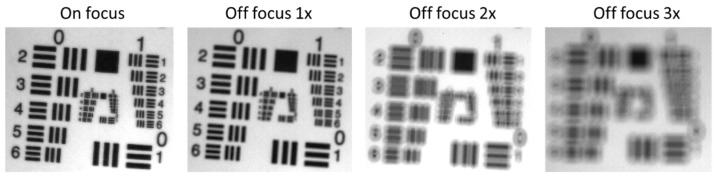
USAF 1951 target images for C-1, showing decreasing focus from left to right.

Camera focus is a critical parameter in achieving optimal spatial resolution. In this study, we examined the effect of focus on spectral data by measuring $I_{w}$ and I at different focus levels for both the HSICs and HSIEs. The extent of off-focus levels was subjectively defined by visualizing the images of the 1951 USAF resolution target. Figure 3 shows different focus levels for C-1. Other HSI hypercubes were captured under similar focus levels. Reflectance in ideal scenarios was calculated when $I_{w}$ and I were measured at the same focus level. Reflectance in realistic scenarios was calculated when $I_{w}$ were measured at the optimal focus (on focus), while I was measured at an off-focus level. Other conditions, such as WD, remained constant, though for C-1, the WD also slightly changed by adjusting the focus due to the zoom lens structure.

#### 2.3.6. Working Distance (WD)

Depending on the application, WD can vary and influence imaging performance, particularly for in vivo tissue, where the surface is not flat and both the tissue and camera may move [33]. The optimal focal distances were 150 mm for C-1 and 90 mm for C-2. The effect of WD was evaluated over a range of 135–165 mm for C-1 and 75–105 mm for C-2, with 10 mm increments. For E-1 and E-2, their optimal focal distance was 10 mm, and the effects of their WD were evaluate over a range of 6–14 mm, with 2 mm increments. The target was mounted on a stage allowing precise vertical WD adjustment, while the camera and light remained fixed (Figure 1). Consequently, illumination intensity, illumination angle, and camera focus could change slightly with WD. Reflectance spectra were calculated under two scenarios: (1) ideal—$I_{w}$ and I were measured at the same WD; and (2) realistic—$I_{w}$ was measured at the default WD while I was measured at varying WDs.

#### 2.3.7. Illumination Angle ($\theta_{ill}$)

$\theta_{ill}$ is important in HSI as it affects the illumination uniformity and surface reflectance. We examined its impact on C-1 and C-2 in Phase 1 using the TH light source. The impact on E-1 and E-2 were not studied since their $\theta_{ill}$ was fixed at 0°. For C-1 and C-2, $\theta_{ill}$ was varied from 30° to 70° in 10° increments. Reflectance spectra were calculated under two scenarios: (1) ideal—both $I_{w}$ and I were measured at the same $\theta_{ill}$, and (2) realistic—$I_{w}$ was acquired at a fixed $\theta_{ill}$ of 30°, while I was measured at varying $\theta_{ill}$.

#### 2.3.8. Target Angle ($\theta_{tar}$)

$\theta_{tar}$ is defined as the angle between the target surface norm and the central axis of field of view (identical to the optical axis in well-designed optics). It can vary by application, particularly in medical imaging where tissue surface is irregular and the imaging system may move. We examined the impact of $\theta_{tar}$ on reflectance spectra across −30° to +30° in 10° increments. Positive and negative $\theta_{tar}$ correspond to counterclockwise and clockwise rotations, respectively (Figure 2). Reflectance spectra were calculated under two scenarios: (1) ideal—$I_{w}$ and I were measured at the same $\theta_{tar}$; and (2) realistic—$I_{w}$ was measured at $\theta_{tar}$ = 0° while I was collected at varying $\theta_{tar}$. For C-2, 80 × 80 pixels were averaged to calculate reflectance spectra by default, but 50 × 50 pixels were used for at $\theta_{tar}$ = ±30°, since the cross-sectional area perpendicular to the optical axis was smaller at these angles, resulting in a reduced target area in the images.

#### 2.3.9. Measurement Repeatability

Measurement repeatability [34] is crucial to ensure that measured reflectance spectra remain consistent when obtained using the same experimental setup. Good repeatability indicates system stability and reliability. To assess repeatability for E-1 and E-2, hypercubes were captured at WD of 8 mm and 10 mm across three rounds under two scenarios: (1) ideal—both $I_{w}$ and I were measured at the same WD; and (2) realistic—$I_{w}$ was measured at 10 mm and I at 8 mm. In each round, E-1 and E-2 were dissembled by removing the systems from the post, separating the endoscope and camera, and then reassembling them. The target platform was also dissembled and reassembled between rounds.

## 3. Results

Phase 1 results demonstrated the effects of various parameters on the spectral performance of C-1 and C-2 using the Red target and TH light source; Phase 2 further examined the impact of different parameters on E-1 and E-2 using the EO target and the Xe light source. The parameters studied in each phase are listed in Table 2. The R and scaled R′ were compared with the Ref under both ideal and realistic scenarios.

### 3.1. Ambient Light

**Figure 4:**
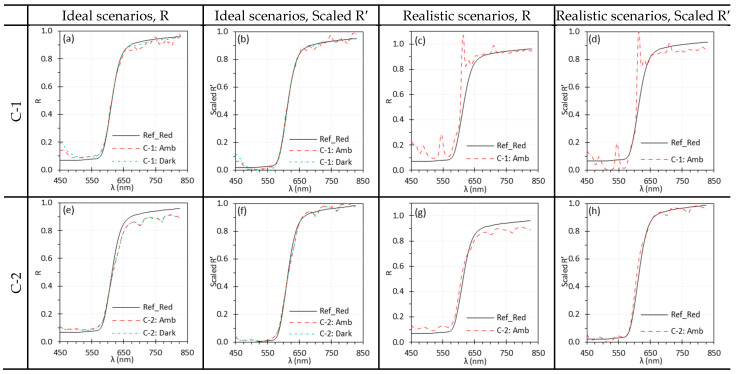
Impact of ambient light on the spectral performance of C-1 (**a**–**d**) and C-2 (**e**–**h**) using the Red target under ideal (**a**,**b**,**e**,**f**) and realistic (**c**,**d**,**g**,**h**) scenarios. (Ref_Red: Ref of the Red target; other legends indicate whether I was measured with ambient light).

Figure 4 illustrates the impact of ambient light on the spectral performance of C-1 and C-2. Under ideal conditions, ambient light had no measurable effect on C-2 (Figure 4e,f), while C-1 showed only minor differences, with slightly increased noise in the 670–830 nm range (Figure 4a,b). The wave-shape artifact in the spectra observed for C-2, which is also visible in other C-2 figures, was later confirmed to be an artifact of the lens. Under realistic scenarios, however, C-1 exhibited pronounced spectral artifacts (Figure 4c,d), whereas C-2 was slightly affected only in the 450–550 nm and 650–850 nm ranges (Figure 4g,h). The higher number of spectral bands and greater spectral resolution of C-1 made it more sensitive to ambient light with sharp spectral peaks. The fluorescent light sources in the laboratory, as illustrated in Section 4.2 generated these sharp spectral peaks in Figure 4c,d. While Scaled R′ improved agreement with reference spectra, normalization could not fully eliminate the artifacts introduced by ambient light.

**Figure 5:**
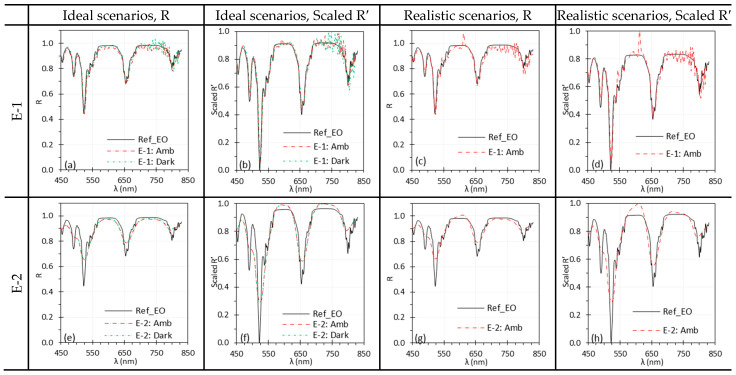
Impact of ambient light on the spectral performance of E-1 (**a**–**d**) and E-2 (**e**–**h**) using the EO target under ideal (**a**,**b**,**e**,**f**) and realistic (**c**,**d**,**g**,**h**) scenarios. (Ref_EO: Ref of the EO target; other legends indicate whether I was measured with ambient light).

Figure 5 shows the impact of ambient light on spectral performance of E-1 and E-2, with trends similar to those observed for C-1 and C-2 in Figure 4. Because of its lower spectral resolution, E-2 failed to capture some sharp peaks present in EO reference spectra (Figure 5e–h), a limitation consistently observed throughout the study. Under realistic scenarios, both E-1 and E-2 exhibited artifacts in the reflectance spectra, especially around 611 nm (Figure 5c,d,g,h) due to the strong peak of ambient fluorescent light (Section 4.2). While the artifact was negligible for the simple Red spectrum captured by C-2 (Figure 4g,h), it was noticeable for the EO spectra captured by E-2 (Figure 5g,h). The artifact peaks were sharper in E-1 because of its higher spectral resolution, while in E-2 they remained noticeable but less sharp. Normalization (Scaled R′) improved overall agreement but did not eliminate the discrepancies (Figure 5d,h), underscoring the need to carefully account for ambient illumination.

### 3.2. Exposure Time

**Figure 6:**
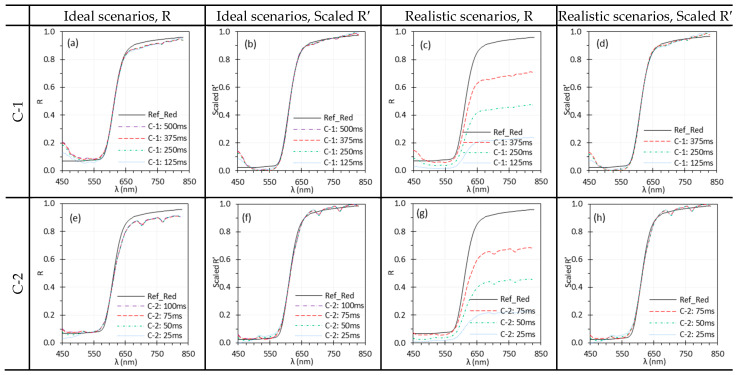
Impact of exposure time on the spectral performance of C-1 (**a**–**d**) and C-2 (**e**–**h**) using the Red target under ideal (**a**,**b**,**e**,**f**) and realistic (**c**,**d**,**g**,**h**) scenarios. (Ref_Red: Ref of the Red target; other legends indicate the exposure time for I measurement).

**Figure 7:**
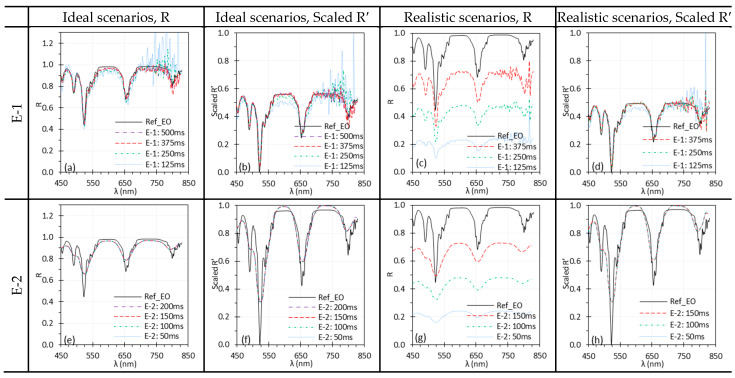
Impact of exposure time on the spectral performance of E-1 (**a**–**d**) and E-2 (**e**–**h**) using the EO target under ideal (**a**,**b**,**e**,**f**) and realistic (**c**,**d**,**g**,**h**) scenarios. (Ref_EO: Ref of the EO target; other legends indicate the exposure time for I measurement).

Figure 6 and Figure 7 illustrate the influence of exposure time on HSICs and HSIEs. In ideal scenarios, exposure time had little impact, but in realistic scenarios it led to a linear change in reflectance. Figure 6c,g show that when $I_{w}$ was collected at higher exposure time than I, the resulting reflectance spectra had lower values than the reference; however, normalization (Figure 6d,h) brought these spectra into close agreement. For C-1, the discrepancies below 450 nm were likely due to the low reflectance of the Red target, weak illumination of TH light, and stray light [35]. This observation remained consistent across all other investigated parameters.

For E-1, shorter exposure times introduced higher noise, particularly in the 700–830 nm range (Figure 7). This reflects the combined effects of reduced SNR at low exposures, sharp Xe light source peaks in this range, and the high spectral resolution of the HSI systems like C-1 and E-1, which divides the signal across many bands. Similar patterns were observed across other parameters.

### 3.3. Camera Warm-Up Time

**Figure 8:**
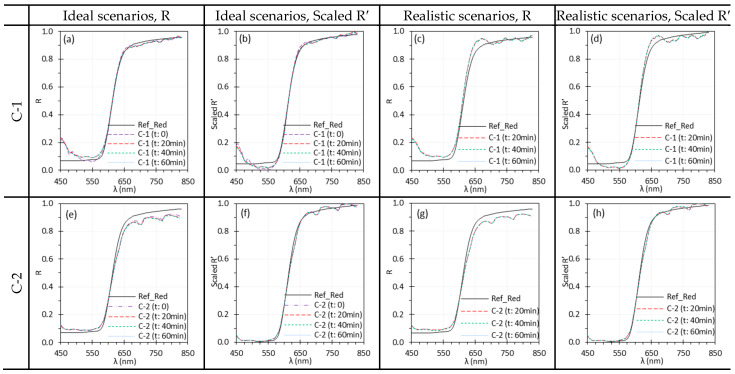
Influence of camera warm-up time on the spectral performance of C-1 (**a**–**d**) and C-2 (**e**–**h**) using the Red target under ideal (**a**,**b**,**e**,**f**) and realistic (**c**,**d**,**g**,**h**) scenarios. (Ref_Red: Ref of the Red target; other legends indicate the camera warm-up time for I measurement).

**Figure 9:**
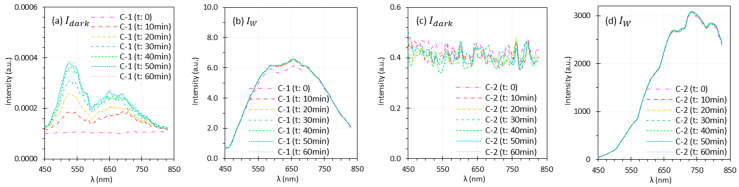
Influence of camera warm-up time on spectra of $I_{dark}$ and $I_{w}$ captured by C-1 (**a**,**b**) and C-2 (**c**,**d**). The legends indicate the camera warm-up time.

Figure 8 illustrates the performance of C-1 and C-2 over a 60-min warm-up period, with spectra displayed at 20-min intervals. Figure 9 presents the raw intensity data ($I_{w}$ and $I_{dark}$) for C-1 and C-2 during the warm-up period, as obtained from each camera’s software. Comparisons should be made within each system, since absolute intensity levels may differ between systems.

For both C-1 and C-2, R spectra overlapped across scenarios (Figure 8a,c,e,g). This is expected under ideal scenarios since $I_{w}$ and I were captured almost simultaneously. Under realistic scenarios, the overlapped spectra indicate that the measured I were relatively stable after ~20 min of warm-up time. However, under realistic scenarios, the spectra of C-1 (Figure 8c,d) appear distorted because reflectance was calculated using $I_{w}$ from time 0. The camera response, and hence the resulting $I_{w}$, changed noticeably in the first 20 min of warm up, especially within the first 10 min (Figure 9b). This issue was observed only for C-1, whose sensor temperature rose sharply from 30.5 °C to 54.3 °C (30.5, 48.5, 51.8, 53.0, 53.3, 54.3, and 54.3 °C at 10-min intervals) during warm-up. Although $I_{dark}$ of C-1 also changed noticeably, its magnitude was negligible compared to $I_{w}$ (Figure 9a). In contrast, C-2 showed only moderate temperature increase of 39–42 °C (39, 40, 41, 41, 42, 41, 40 °C at 10-min intervals) over the same period.

### 3.4. Spatial and Temporal Averaging

**Figure 10:**
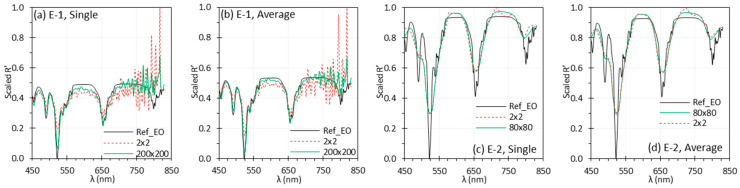
Influence of spatial and temporal averaging on E-1 (**a**,**b**) and E-2 (**c**,**d**) with (**b**,**d**) and without (**a**,**c**) temporal averaging using the EO target. (Ref_EO: Ref of the EO target; other legends indicates ROI size).

Figure 10 shows the Scaled R′ from E-1 and E-2 using spatial averaging over ROI of 2 × 2 and 200 × 200 pixels, with and without temporal averaging. The corresponding root mean square error (RMSE) values between pixels in the region, are shown in Table 3. Both larger ROIs and temporal averaging improved spectral accuracy, though the effect of temporal averaging was smaller. For E-1, increasing ROI size markedly improved spectral accuracy. In contrast, E-2 showed little benefit from spatial averaging, aside from a slight deviation at 700–750 nm with small ROIs that was corrected by larger ROIs. Overall, spatial averaging had a greater impact on E-1 than on E-2.

### 3.5. Camera Focus

**Figure 11:**
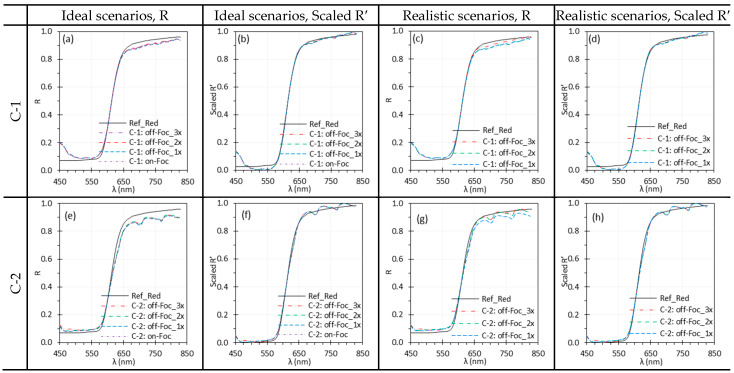
Impact of camera focus on the spectral performance of C-1 (**a**–**d**) and C-2 (**e**–**h**) using the Red target under ideal (**a**,**b**,**e**,**f**) and realistic (**c**,**d**,**g**,**h**) scenarios. (Ref_Red: Ref of the Red target; other legends indicate the focus level (Figure 3) for I measurement).

**Figure 12:**
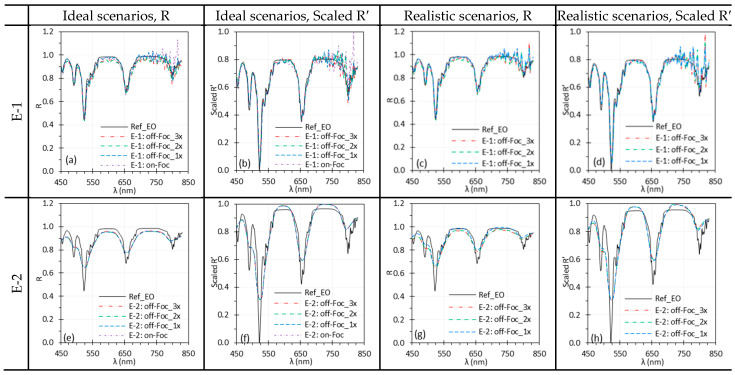
Impact of the camera focus on the spectral performance of E-1 (**a**–**d**) and E-2 (**e**–**h**) using the EO target under ideal (**a**,**b**,**e**,**f**) and realistic (**c**,**d**,**g**,**h**) scenarios. (Ref_EO: Ref of the EO target; other legends indicate the focus level (Figure 3) for I measurement).

Figure 11 and Figure 12 show the influence of camera focus on spectral performance, comparing best focus (i.e., on-Foc) with three levels of off-focus (off-Foc), with 3× representing the worst focus condition (Figure 3). Focus changes slightly altered WD for C-1 but not for C-2, E-1, or E-2.

Under ideal scenarios for C-1, C-2 and E-2, focus variations had little impact on reflectance spectra (Figure 11a,b,e,f and Figure 12e,f). In realistic scenarios, some discrepancies appeared across focus levels for C-1, C-2 and E-2 (Figure 11c,g and Figure 12g), but these differences were small and largely corrected in the Scaled R′ spectra. As in Figure 7, E-1 spectra were noisier (Figure 12a–d), especially beyond 750 nm, with a pronounced peak near 820 nm likely due to sharp peaks in the Xe light source emissions (Section 4.2).

### 3.6. Working Distance

**Figure 13:**
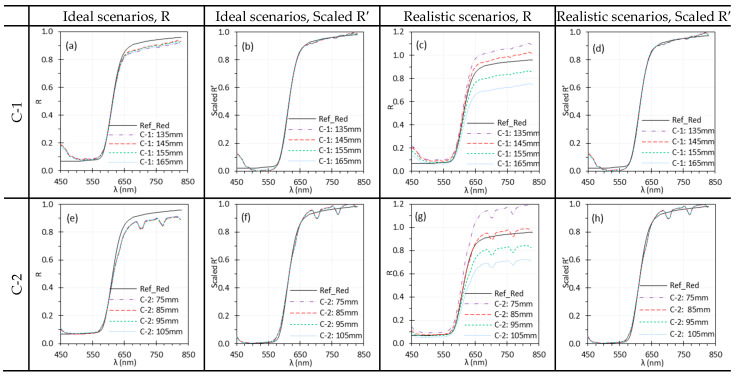
Impact of WD on spectral performance of C-1 (**a**–**d**) and C-2 (**e**–**h**) using the Red target under ideal (**a**,**b**,**e**,**f**) and realistic (**c**,**d**,**g**,**h**) scenarios. (Ref_Red: Ref of the Red target; other legends indicate the WD for I measurement).

**Figure 14:**
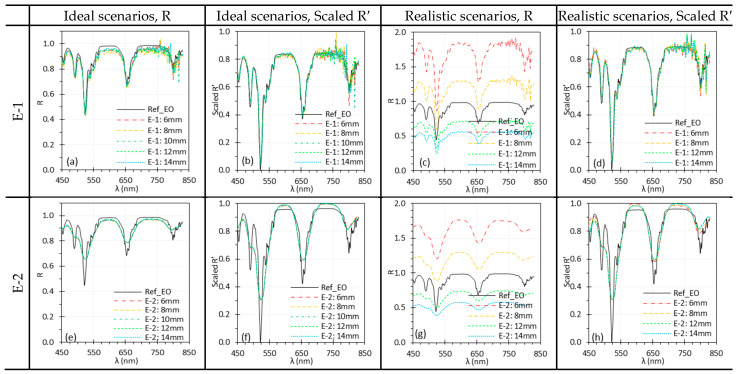
Impact of WD on the spectral performance of E-1 (**a**–**d**) and E-2 (**e**–**h**) using the EO target under ideal (**a**,**b**,**e**,**f**) and realistic (**c**,**d**,**g**,**h**) scenarios. (Ref_EO: Ref of the EO target; other legends indicate the WD for I measurement).

Figure 13 and Figure 14 show the impact of WD on spectral performance of the HSICs and HSIEs. In ideal scenarios, minor discrepancies in C-1 and E-1 spectra (Figure 13a and Figure 14a) were minimized through normalization (Figure 13b and Figure 14b). The spectra for C-2 and E-2 overlapped in ideal scenarios. However, in realistic scenarios, WD significantly influenced the spectra (Figure 13c,g and Figure 14c,g). While normalization reduced these discrepancies, slight differences remained for E-1 and E-2 compared to C-1 and C-2. Nevertheless, the differences were negligible, and the spectral locations of the peaks remained consistent.

### 3.7. Illumination Angle

**Figure 15:**
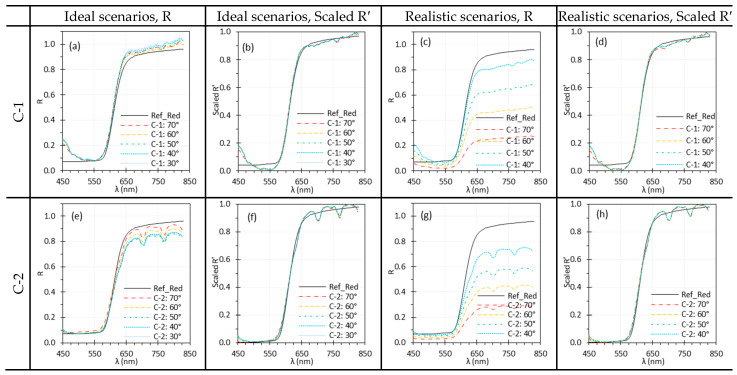
Impact of $\theta_{ill}$ on the spectral performance of C-1 (**a**–**d**) and C-2 (**e**–**h**) using the Red target under ideal (**a**,**b**,**e**,**f**) and realistic (**c**,**d**,**g**,**h**) scenarios. (Ref_Red: Ref of the Red target; other legends indicate the $\theta_{ill}$ for I measurement).

Figure 15 demonstrates the impact of $\theta_{ill}$ on the spectral performance of C-1 and C-2. Under ideal scenarios, only minor discrepancies were observed, likely due to slight misalignment of the target rotation axis with the rotating stages, which can affect WD for $I_{w}$ and I measurement. In realistic scenarios, the reflectance spectra varied significantly with $\theta_{ill}$, but normalization effectively minimized these discrepancies.

### 3.8. Target Angle

**Figure 16:**
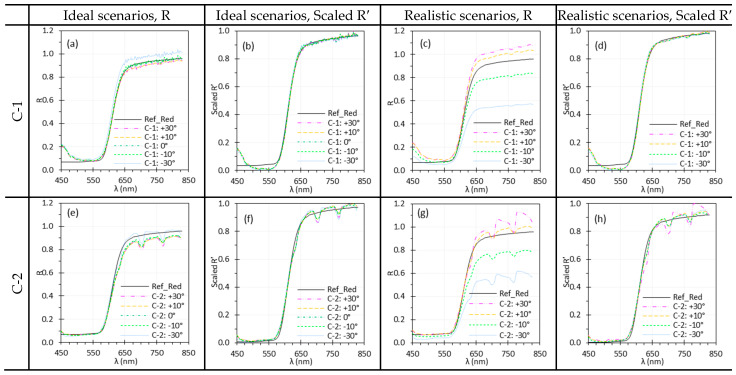
Impact of $\theta_{tar}$ on the spectral performance of C-1 (**a**–**d**) and C-2 (**e**–**h**) using the Red target under ideal (**a**,**b**,**e**,**f**) and realistic (**c**,**d**,**g**,**h**) scenarios. (Ref_Red: Ref of the Red target; other legends indicate the $\theta_{tar}$ for I measurement).

**Figure 17:**
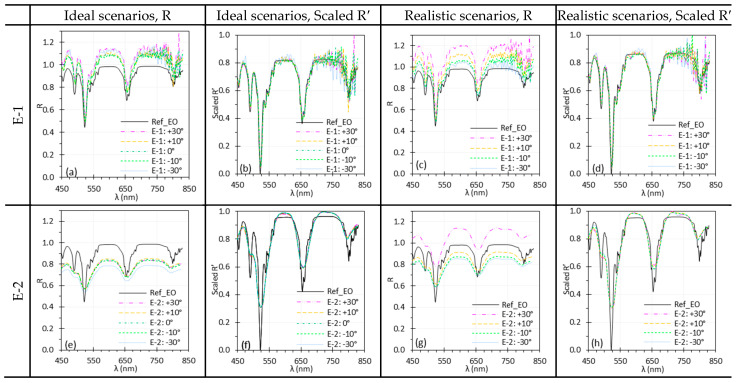
Influence of $\theta_{tar}$ on the spectral performance of E-1 (**a**–**d**) and E-2 (**e**–**h**) using the EO target under ideal (**a**,**b**,**e**,**f**) and realistic (**c**,**d**,**g**,**h**) scenarios. (Ref_EO: Ref of the EO target; other legends indicate the $\theta_{tar}$ for I measurement).

Figure 16 and Figure 17 show the influence of $\theta_{tar}$ on the spectral performance of HSICs and HSIEs. Similar to $\theta_{ill}$, minor discrepancies between spectra acquired at different $\theta_{tar}$ appeared under ideal scenarios, while larger ones emerged under realistic scenarios due to variations in light intensity and uniformity across the target surface as $\theta_{tar}$ changed. Most discrepancies were minimized through normalization, except for C-2 at 30°, where residual differences likely resulted from the smaller ROI (50 × 50 pixels) used for spectral analysis.

### 3.9. Measurement Repeatability

**Figure 18:**
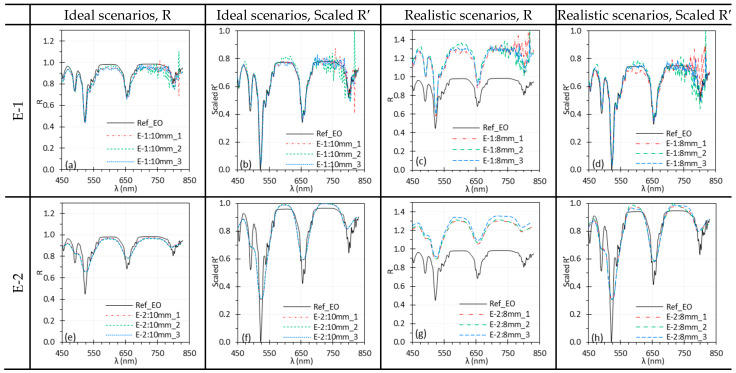
Spectral measurements of E-1 (**a**–**d**) and E-2 (**e**–**h**) using the EO target at WD of 8 mm and 10 mm, each measured over three rounds, under ideal (**a**,**b**,**e**,**f**) and realistic (**c**,**d**,**g**,**h**) scenarios. (Ref_EO: Ref of the EO target; other legends indicate the WD and the round numbers for each I acquisition).

To investigate the repeatability of E-1 and E-2, EO target reflectance was measured at WDs of 8 and 10 mm across three rounds (Figure 18). For E-1, the spectra under both ideal and realistic scenarios exhibited modest differences, primarily due to spectral noise in the 680–830 nm range caused by the Xe light source peaks (Section 4.2). In contrast, E-2 spectra overlapped under ideal conditions and showed only slight variations under realistic scenarios, likely due to minor WD shifts during the disassembly and reassembly. Normalization further reduced these discrepancies. Overall, both E-1 and E-2 demonstrated good repeatability under both ideal and realistic conditions.

## 4. Discussion

Identification and mitigation of artifacts associated with system configuration and experimental parameters can improve the spectral accuracy of HSI systems. In our previous study [25], we evaluated the efficacy of nine normalization methods for improving the robustness of reflectance spectra acquired by HSI systems. In the current study, we investigated the effects of eight system configuration and experimental parameters on reflectance spectra captured by two HSICs (Phase 1) and two HSIEs (Phase 2).

### 4.1. HSI Technologies

Two distinct HSI technologies were employed in this study. C-1 and E-1 use spectral scanning via a Fabry–Perot interferometer, while C-2 and E-2 use snapshot technology. Each technology presents distinct trade-offs, enabling HSI technology to be tailored to specific applications across diverse fields. Spectral scanning offers hypercubes with high spatial and spectral resolution but requires long acquisition times and is more susceptible to external parameters such as illumination light. In this study, the reflectance spectra of the EO target captured by the spectral scanning technology are more accurate but noticeably nosier than those captured by the snapshot technology. We avoided software averaging to prevent loss of subtle spectral features. In contrast, the rapid acquisition capability with snapshot imaging appears advantageous for real-time applications; however, spectral and spatial resolution are limited [9,36], as each spectral band utilizes only a small portion of the sensor. This limitation may reduce a system’s ability to detect fine spectral or spatial features associated with diseases. In this study, some small spectral features of the EO target were missed in the reflectance spectra captured by E-2. In summary, spectral scanning provided more detailed functional information, while snapshot imaging offers greater robustness and operational stability.

### 4.2. Illumination Spectra

**Figure 19:**
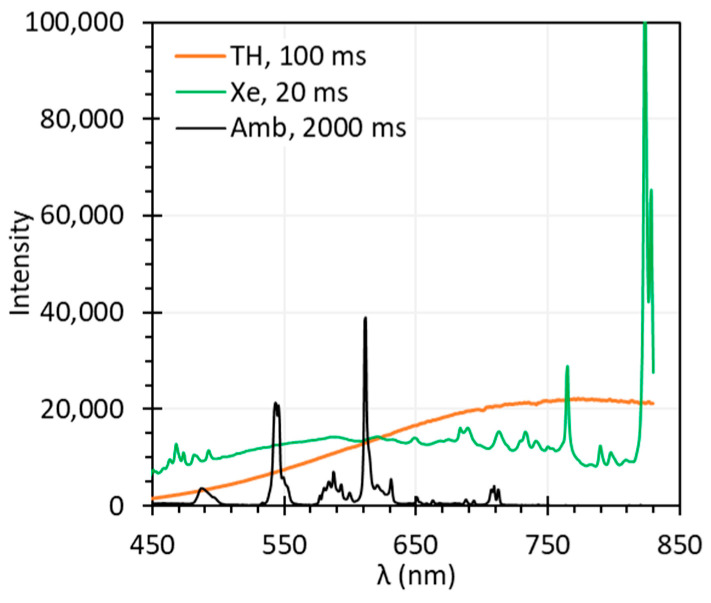
Spectra of the TH light source, Xe light source and ambient light at different exposure times.

Illumination spectra, primarily determined by the HSI system’s light source and influenced by ambient light, are crucial for accurate reflectance measurement. To assess their impact, we measured the spectral outputs of the TH light source, Xe light source, and ambient light (Figure 19) using a spectrometer (QE65 Pro, Ocean Optics, Orlando, FL, USA). The relative intensity of these spectra can be interpreted based on their exposure time; however, the absolute intensity values lack direct significance for HSI due to variations in instrument sensitivity, detector efficiency, measuring distance, and exposure time.

The spectrum of the TH light source was smooth, but the intensity was low in the blue/UV range, limiting sensitivity in shorter wavelengths critical for certain diagnostic tasks. If a target exhibits low reflectance below 500 nm, the resulting reflectance spectra under TH light source illumination may suffer from a low SNR and become susceptible to stray light [35], leading to deviations from true values. This effect was evident in the reflectance spectra of the Red target captured by C-1 (Figure 6a,b). The spectra captured by C-2 did not show significant deviation, because C-2 has lower spectral resolution thus wider bandwidth, resulting in higher intensity within each band compared to C-1. Another challenge for TH light sources is that they are inefficient and may generate excessive heat, which might cause patient discomfort or tissue damage. They also have limited lifespan.

Compared with the TH light source, the Xe light source provides approximately five times higher intensity across the 450–830 nm range, with sharp peaks near 764 nm and 823 nm, and smaller peaks in the 450–500 nm and 680–830 nm ranges. These peaks can introduce noise in the spectra captured by a HSI system with high spectral resolution, as seen in the EO spectra captured by E-1. In our previous study, our results indicated that the reflectance spectra of the same EO target captured by C-1 were much noisier under Xe illumination than under TH illumination [25].

The ambient light had much lower intensity than the TH and Xe light sources. However, the sharp spectral peaks at 487 nm, 545 nm, 587 nm, and 611 nm introduced artifacts into the reflectance spectra, especially when using a high-resolution HSI systems such as C-1 and E-1. Figure 4c and Figure 5c illustrate the artificial peaks caused by the ambient light, especially around the 545 nm and 611 nm, under realistic scenarios where $I_{w}$ and I were measured under different ambient light conditions. C-2 and E-2 showed slightly fewer discrepancies under the ambient light, possibly because their lower spectral resolution prevents them from capturing such sharp peaks.

In summary, the optimal light source for HSI should exhibit a spectrally uniform output—characterized by a smooth, flat spectrum—with sufficient intensity across the desired wavelength range. Sharp peaks in the light source or ambient light can introduce artifacts in the reflectance spectra. If ambient light is spectrally uniform and $I_{w}$ and I are measured under the same conditions, its effect on HSI is negligible. Otherwise, ambient light should be minimized or avoided. While not specifically discussed in this paper, the warm-up of the light source is also important. Insufficient light source warm-up time can lead to unstable spectral output and inconsistent spectral intensity. Additionally, this study did not address photobiological safety considerations for human exposure, which should be assessed in accordance with applicable standards such as IEC 62471, IEC 60825-1, or ANSI Z136.1 [37,38,39], depending on the type of light source used, particularly in the context of clinical applications.

### 4.3. Spectral Noise

Noise in the spectra can come from an unstable sensor or low SNR. Low exposure time or illumination intensity decreases SNR and introduces noise into the reflectance spectra. Figure 6 and Figure 7 illustrate the impact of exposure time on the spectral performance of HSIC and HSIE systems, respectively. For all systems under realistic scenarios, spectra without normalization exhibit significant discrepancies due to variations in exposure time, while normalization effectively mitigated these effects. For E-1, most spectra were highly unstable and noisy beyond 680 nm (e.g., Figure 10a,b), although some spectra demonstrated acceptable accuracy in this range (e.g., Figure 5a,b). This was likely due to two factors: (1) E-1’s higher spectral resolution with narrower spectral bands reduced light intensity per band compared to E-2; and (2) the Xe light source produced a noisy spectrum, especially above 680 nm, which had a greater effect on E-1 than E-2. Since SNR is approximately proportional to exposure time under a fixed illumination intensity, shorter exposure time results in lower SNR, as observed in the E-1 spectra in Figure 7. However, longer exposure time may introduce artifacts such as blurring and degraded reflectance spectra due to motion, particularly with unsecured HSI setups or moving tissues, offsetting the benefits of increased SNR. Increasing illumination intensity can reduce the required exposure time and improve SNR, thereby enhancing spectral accuracy, provided the tissue damage threshold is not exceeded.

A stable sensor needs sufficient camera warm-up time. Figure 8 and Figure 9 illustrate the impact of camera warm-up time on performance. Changes in $I_{dark}$ and $I_{w}$ were apparent for C-1 (Figure 9a,b) but minimal for C-2 (Figure 9c,d), corresponding to sensor temperature increase of about 13 °C for C-1 and only 3 °C for C-2 over 60 min. Notably, the change of $I_{w}$ in the first 10 min was significant for C-1, leading to the artifacts in reflectance spectra (Figure 8c,d). These artifacts persisted even after normalization. It should be noted that the required warm-up time may vary between different cameras.

Spatial and temporal averaging reduces spectral noise, and appropriate ROI size is crucial to minimize spatial SNR variation and suppress outliers. For each spectral band, 200 × 200 pixels were averaged for C-1/E-1 and 80 × 80 pixels for C-2/E-2. As shown in Figure 10, using a smaller ROI (2 × 2 pixels) greatly increased noise in E-1, while E-2 showed minimal difference between ROI sizes. Averaging over a large ROI improved SNR, reduced the influence of outliers, and yielded more stable spectral measurements, especially in homogeneous regions. However, this came at the cost of reduced spatial resolution and the potential mixing of distinct spectral signatures in heterogeneous areas. Careful ROI selection is therefore critical to balance noise reduction with spectral fidelity.

In this study, reflectance spectra were calculated by averaging three measurements to reduce temporal variance. As shown in Figure 10, temporal averaging slightly reduced spectral noise but was less effective than increasing the ROI size. This is primarily because only three measurements were used for temporal averaging, whereas thousands of pixels contributed to spatial averaging. Although temporal averaging can slightly improve spectral quality by reducing random noise, it increases acquisition time and may introduce motion artifacts in dynamic scenes (e.g., blood flow, body motion) or when the HSI setup is unstable. For high-resolution spatial or spectral scanning HSI systems like C-1 and E-1, temporal averaging is impractical for in vivo measurements and therefore not suitable for bench testing.

### 4.4. Focus and Working Distance

Camera focus is directly related to spatial resolution. A well-focused HSI camera captures spectral signals from precise locations, whereas an out-of-focus camera collects signals from a broader area with blurred boundaries. Figure 11 and Figure 12 depict the effect of camera focus on the spectral performance of HSICs and HSIEs, respectively. No significant differences were observed across all scenarios, primarily because the Red and EO targets are homogenous, so spatial information did not impact spectral accuracy. Slight defocusing can reduce spectral noise, as seen in Figure 12a,b; however, this benefit comes at the cost of reduced spatial resolution. In clinical applications, focus should be carefully controlled: when reflectance spectra from fine tissue structures (e.g., mapping saturation in microvasculature) are required, precise focus is essential, whereas slight defocus may be acceptable when spectral data from a larger area is sufficient.

The HSI systems used in our study did not feature autofocus. Therefore, change in WD affected both magnification and spatial resolution. In our setup, at their respective focal distances of 150 mm and 90 mm, each pixel of C-1 and C-2 corresponded to 0.03 × 0.03 mm^2^ and 0.18 × 0.18 mm^2^ on the targets, respectively. For E-1 and E-2, with a focal distance of 10 mm, each pixel corresponded to 0.006 × 0.006 mm^2^ and 0.04 × 0.04 mm^2^, respectively. In clinical applications, these measurements determine the minimum sample size that can be analyzed without confounding one tissue type with another.

Additionally, since the relative positions between the HSI systems and the light sources are fixed, changing the WD also alters the illumination intensity. Figure 13 and Figure 14 illustrate the impact of WD on the spectral performance of HSICs and HSIEs. Under realistic scenarios, dramatic spectral differences are observed when WD changes (Figure 13c,g and Figure 14c,g), mainly due to variation in illumination intensity. However, these differences were largely mitigated through normalization, as shown in Figure 13d,h and Figure 14d,h. This highlights the importance of normalization in ensuring consistent spectral results despite WD variations.

### 4.5. Illumination and Target Angles

We studied the effects of $\theta_{ill}$ and $\theta_{tar}$ on spectral performance. Changes in $\theta_{tar}$ influence both $\theta_{ill}$ and the image capture angle. Under ideal scenarios, spectra measured at different $\theta_{ill}$ or $\theta_{tar}$ angles showed minor variations (Figure 15a,e and Figure 16a,e), mainly due to alignment issue when switching the White and test (Red or EO) targets. Under realistic scenarios, however, variations were much more pronounced (Figure 15c,g and Figure 16c,g), showing that both $\theta_{ill}$ and $\theta_{tar}$ significantly affected reflectance spectra by altering irradiance uniformity on the target surface. In realistic scenarios, $\theta_{tar}$ affected spectral measurements for both E-1 and E-2 (Figure 17c,g), with the impact depending on the direction of $\theta_{tar}$ rotation. This variation arises from the optical design of rigid endoscopes, which produces non-uniform irradiance across the target surface [40]. Depending on the surface’s reflectance behavior—such as Lambertian, semi-Lambertian, or specular—characterized by the bidirectional reflectance distribution function (BRDF), the resulting scene radiance varies accordingly. This variation, in turn, impacts the irradiance received by the image sensors and thereby alters recorded spectral intensity. These effects, however, can be mitigated using proper normalization methods (Figure 15b,d,f,h and Figure 16b,d,f,h).

Our study focuses on targets with a flat Lambertian surface and a consistent BRDF. If the BRDF varies across different surface locations, or if the surface is uneven—such that oblique illumination introduces shadows and non-uniform shading that distort spatial details and spectral measurements—our conclusion may no longer hold. In addition, changes in angle may result in glare or hotspots caused by direct reflection into the camera, especially from shiny tissue surfaces, which should be avoided.

### 4.6. Normalization Methods

Normalization is critical for accurately assessing spectral performance, as it reduces the influence of external parameters and ensuring that comparisons reflect target-specific characteristics. In our previous study, we evaluated nine normalization methods and found that most performed similarly, except for the Max and MinMax methods, which were less reliable for spectra with sharp noise peaks [25]. The methods involving linear transformations across the entire spectrum—such as AUC, Sum, Ave, L2, SNV, and Pareto—were generally robust against outliers and noise, as they incorporate reflectance values across all wavelengths to generate a composite measure. Among these, the SNV method demonstrated slightly better performance for our data. Consequently, we applied the SNV normalization followed by a uniform scaling step in this study to convert raw reflectance (R) spectra into Scaled R′ spectra. This does not imply that other methods are unsuitable; the choice of normalization method should be guided by spectral characteristics, data quality, and the intended analysis algorithm. Under different conditions, other methods may yield comparable results.

Exposure time, working distance, illumination angle, and target angle can significantly affect the reflectance intensity. In endoscopic imaging, tissues are rarely flat, so different regions of the field of view inevitably lie at different distances and angles relative to the endoscope. As a result, distance- and angle-dependent effects are unavoidable, making normalization essential for consistent spectral analysis. Practically, this means that quantitative spectral detection and classification rely primarily on changes in the shape of the spectral curve rather than its magnitude.

While normalization effectively mitigates external influences and enhances spectral comparability, it introduces trade-offs. Specifically, normalization removes information contained in the absolute magnitude of the spectrum (i.e., total intensity), which may reflect important biologically or physically relevant features such as tissue density, the presence of broad absorbers, blood pooling, or variations in surface reflectance. Thus, although normalization is often necessary in clinical applications, the potential loss of magnitude-related information should be carefully considered and acknowledged as a limitation.

### 4.7. Study Limitations

The primary purpose of HSI in medical applications is to reveal the composition, structure, and properties of biological tissue by measuring wavelength-dependent reflectance or fluorescence signals. These signals are governed by tissue absorption, scattering, and fluorescence characteristics, but can also be influenced by external factors. This study represents a systematic effort to quantify how such factors affect HSI performance under controlled experimental conditions. While this approach enabled us to isolate and quantify the impact of each parameter, several limitations must be acknowledged.

Scope of parameters studied. This work specifically evaluated the influence of eight system configuration and experimental parameters on HSI performance in benchtop experiments. Other important spectral and spatial performance characteristics—such as the spectral response function (SRF), image distortion, noise, and modulation transfer function [35,41,42]—were outside the scope of this study and may be addressed in future work.

HSIE technologies and reproducibility. This study employed off-the-shelf HSI detectors with a rigid endoscope to provide a mechanically simple and reliable HSIE for assessing various parameters. In clinical practice, however, an HSIE may incorporate diverse platforms (rigid, flexible, capsule-based) and detector configurations (push-broom, spectral scanning, interferometric, snapshot), each with inherent advantages and limitations. For example, snapshot detectors can enable high-speed or real-time imaging but often at the expense of reduced spectral resolution [9,12,43]. A thorough comparison of detector configurations is beyond the scope of this work, but the parameters evaluated here form an initial framework for characterization of HSIEs—an important step toward enabling cross-system comparisons and optimization. In addition, only one camera was evaluated for each modality (spectral scanning and snapshot). While the observed differences can be reasonably attributed to underlying technology, caution is warranted in extending these findings to other cameras, even within the same modality, as variations in design, calibration, and acquisition protocols may also affect spectral accuracy. More broadly, reproducibility across HSI systems and vendors remains a critical challenge. Multi-system and multi-site evaluations will be essential to ensure consistent performance and support reliable clinical deployment.

Clinical perspective. Although our findings provide a foundation for understanding parameter sensitivity in HSI, their direct clinical applicability remains to be established. The technical characteristics were evaluated solely in benchtop experiments with homogeneous Lambertian targets, without validation on biological tissues. In clinical settings, additional factors such as tissue motion, blood flow, uneven surfaces, and dynamic illumination can introduce variability beyond the targets utilized here. Thus, the results should be interpreted as benchtop system-level assessments rather than evidence of clinical performance. Future studies incorporating non-ideal conditions and biological specimens or tissue-simulating phantoms [44] will strengthen clinical relevance and facilitate translation.

Normalization approaches. Our study applied a single normalization approach (SNV with uniform scaling), which was selected based on our prior comparative analysis that found it to be robust across bench tests. However, we acknowledge that other approaches (e.g., physics-based modeling, hybrid correction algorithms, multiplicative scatter correction, derivative [45]) may yield different outcomes. Further evaluation of these advanced techniques is necessary. In our previous study [25], we employed a two-stage approach to investigate the efficacy of different normalization methods, which can be extended in the future to examine other types of normalization and preprocessing methods.

Spectral noise analysis. While we noted that normalization was ineffective at eliminating noise under low exposure times or small ROI sizes, this work did not explicitly dissect the physical sources of noise (e.g., photon shot noise, readout noise, spectral misregistration) [46]. Nor did we evaluate mitigation strategies such as denoising algorithms, adaptive exposure control, or advanced reconstruction techniques [47]. Addressing these issues represents an important next step in improving HSI data fidelity.

Taken together, these limitations underscore that our study represents a fundamental step toward building a rigorous framework for optimizing HSI systems. By systematically identifying how fundamental parameters affect spectral accuracy under controlled conditions, we provide groundwork that can be expanded to additional performance metrics, more advanced normalization strategies, noise modeling, and ultimately, clinical validation.

## 5. Conclusions

We systematically evaluated the influence of eight system configurations and experimental parameters on the spectral performance of four representative HSI systems (C-1, C-2, E-1, E-2) under both ideal and realistic scenarios, and assessed how normalization mitigate these effects. Parameters such as ambient light, exposure time, camera warm-up time, spatial and temporal averaging, camera focus, working distance, and angular configurations (illumination and target angles) each introduced varying degrees of degradation in spectral performance. Among these, geometric changes, such as focus, working distance, and illumination and target angles, introduced discrepancies that were generally mitigated through normalization. Ambient light, exposure time, and spatial averaging had the most pronounced effects, especially for system configurations with high spectral resolution (e.g., C-1 and E-1), due to their sensitivity to signal variability and spectral peaks in the illumination. Camera warm-up also impacted C-1 performance due to greater thermal drift. Normalization improved comparability across conditions but did not fully compensate for all discrepancies, underscoring the importance of consistent acquisition protocols to ensure accurate and reliable spectral measurements in practical settings.

To our knowledge, this study represents the first comprehensive analysis of eight key parameters across both interferometer-based spectral scanning and snapshot HSI techniques. By integrating parameter sensitivity analysis with an evaluation of normalization strategies, we establish a framework for characterizing HSI systems and identifying best practices to minimize measurement errors. Table 4 summarizes the parameters studied and offers suggestions to minimize errors in reflectance measurement. These insights may help optimize HSI system performance in medical applications, enhancing diagnostic reliability and clinical utility.

Although our findings are based on benchtop experiments with homogeneous Lambertian targets, they underscore the importance of optimizing acquisition conditions and normalization approaches to achieve reproducible and accurate reflectance spectra. Such reproducibility is essential for consistent tissue classification and diagnostic decision-making, where discrepancies across devices and acquisition conditions could undermine reliability. Future work incorporating biological tissues and dynamic clinical environments will be critical to validate these results and extend their translational relevance. Collectively, these insights advance HSI toward clinical deployment by guiding system optimization and supporting the generation of reliable spectra across diverse platforms and settings.

## Figures and Tables

**Table 1:** Equipment list.

Components	Abbreviation	Models	Functions and Specifications
HSI cameras (HSICs)	C-1	4250 VNIR	Spectral scanning with a Fabry–Perot Interferometer (FPI), coupled with a customized manual zoom lens
	C-2	Ultris SR5	Snapshot imaging, using a linear variable filter (LVF), coupled with a customized manual zoom Lens.
Rigid endoscope	-	502-537-010	The main component of an HSIE. Designed for fluorescence imaging, with good transmission within near-infrared range.
Endoscope coupler	-	8020I	22.5–50 mm parfocal zoom, connecting C-1 or C-2 to the rigid endoscope.
Light sources	Xe	Dyonics 300XL	Xenon light source coupled to E-1 and E-2 by default.
	TH	SLS201	Tungsten halogen light source for C-1 and C-2 by default.
HSI endoscopes (HSIEs)	E-1	-	Combination of C-1, the rigid endoscope, the endoscope coupler, and the Xe.
	E-2	-	Combination of C-2, the rigid endoscope, the endoscope coupler, and the Xe.
Targets	White	SRT-99-100	White Spectralon^®^ diffuse reflectance target (99% reflectance across a wide spectral range). To measure $I_{w}$ in Equation (1).
	Red	SCS-RD-010	Red Spectralon^®^ diffuse reflectance target, for C-1 and C-2 by default. To measure I in Equation (1).
	EO	WCS-EO-010	Spectralon^®^ wavelength calibration target with erbium oxide (EO), for E-1 and E-2 by default. To measure I in Equation (1).

**Table 2:** Parameters studied in different phases.

Parameters	Ambient Light	Exposure Time	Camera Warm-Up Time	Spatial & Temporal Averaging	Camera Focus	WD	$\theta_{ill}$	$\theta_{tar}$	Measurement Repeatability
Phase 1: C-1 and C-2	√	√	√	-	√	√	√	√	-
Phase 2: E-1 and E-2	√	√	-	√	√	√	-	√	√

Note: “√” = studied parameter; “-” = not studied parameter.

**Table 3:** RMSE values between pixels within the ROI for spatial and temporal averaging.

ROI Size (Pixels)	E-1, Single	E-1, Average	E-2, Single	E-2, Average
2 × 2	0.089	0.084	0.090	0.086
E-1:200 × 200/E-2:80 × 80	0.048	0.035	0.083	0.082

**Table 4:** Parameters affecting HSI systems: Effects and Considerations.

Parameters	Effects	Considerations
Illumination spectrum (light source + ambient light)	Illumination spectrum can significantly impact spectral accuracy, especially for HSI systems with high spectral resolution. TH light sources offer smooth but weak light at short wavelengths, while Xe light sources provide stronger light with sharp peaks that can introduce artifacts. Ambient light with spectral peaks, though dimmer, can also distort spectra.	An ideal light source for HSI exhibits spectrally uniform output with sufficient intensity. Reduce or eliminate ambient light and assure the source does not contain sharp spectral peaks. $I_{w}$ and I should be acquired under illumination whose spectra overlap after normalization.
Exposure time	Insufficient exposure, which may result from low scene radiance or short exposure time, can lead to elevated noise levels due to low SNR. This issue is particularly pertinent in HSI systems with high spectral resolution (e.g., E-1). While normalization can reduce spectral differences across exposure settings, it cannot compensate for the increased noise under low exposure conditions.	Illuminate the target with sufficient intensity and use an adequate exposure time without saturation to capture high-quality reflectance spectra; apply normalization to mitigate low exposure effects.
Camera warm-up time	C-1 showed significant $I_{w}$ and $I_{dark}$ changes during warm-up, with sensor temperature rising from 30.5 °C to 54.3 °C, causing artifacts in reflectance spectra. In contrast, C-2 had minimal temperature change (39 °C to 42 °C) and relatively stable performance.	Sufficient warm-up time is critical for stable spectral performance, especially for HSI systems with large thermal drift. The required warm-up time may vary by system. Validate for each system to ensure accurate reflectance measurements.
Spatial and temporal averaging	Spatial averaging using a large ROI significantly reduced spectral noise, particularly for C-1 and E-1, but may compromise spatial resolution. Temporal averaging offered only modest noise reduction and can reduce scanning speed.	Match the ROI size to the feature size in realistic scenarios. Use a large ROI only if the target is mostly homogenous and lacks fine spatial detail. Avoid temporal averaging in bench tests if the realistic applications involve dynamic targets/tissue.
Camera focus	Slight defocus had little impact on reflectance spectra and can even slightly reduce noise for a uniform target. However, defocus compromises spatial resolution. Small discrepancies can be mitigated by normalization.	Proper focus is important for preserving spatial resolution. In clinical imaging, precise focus is needed for fine structures, while slight defocus may be acceptable for larger, uniform areas. Apply normalization to mitigate defocus effects.
WD	For HSI systems without autofocus feature, WD changes affect both spatial resolution and illumination intensity. Under realistic conditions, WD changes significantly affected spectra across all tested systems, though normalization largely mitigated the differences, preserving peak locations.	Maintain a relatively consistent WD if possible. Apply normalization to mitigate the effects of WD change.
$\theta_{ill}$ and $\theta_{tar}$	Changes in $\theta_{ill}$ and $\theta_{tar}$ affect irradiance uniformity and scene radiance depending on the target’s BRDF, leading to spectral variations. Normalization can mitigate the spectral difference due to angle change.	Care is needed when imaging surfaces with variable BRDFs or uneven textures. Minimize oblique angles that may cause glare, shadows, or shading artifacts for accurate spectral measurements. Apply normalization to mitigate angle-induced variations.

Note: The effect of ambient light, together with the effect of light source from our previous publication [25], are grouped under illumination spectrum.

## Data Availability

The original contributions presented in this study are included in the article. Further inquiries can be directed to the corresponding author.

## Appendix A

**Table A1:** Characterization of HSI cameras claimed by the manufacturer.

	Characteristics	C-1	C-2
Spectral	Spectral range (nm)	400–1000	450–850
	Number of spectral bands/channels	Up to 301	51
	Spectral sampling interval (nm)	2–50 *	8
	Spectral response function (SRF) peak width	~4 nm	26 nm @ 532 nm
	Spectral accuracy	±0.6 nm RMSE	-
Spatial	Number of output pixels per band	1936 × 1216	290 × 275
	Field of view	15°	15°
Temporal	Exposure time (ms)	0.1–1000	0.1–60,000
	Hypercube rate	One hypercube/~2.5 min (500 ms exposure, 301 bands) **	Maximum 15 Hz
Other	Bit depth (bits)	8 or 16	12
	Dimensions (mm)	78 × 81 × 198	29 × 29 × 107
	Weight (g)	1247	176
	Operating temperature (°C)	15–40	-
	Pixel size (µm)	5.86	3.45

* During all measurements the maximum number of bands with the smallest sampling interval of 2 nm was used. ** Not reported by the manufacturer; estimated during our measurement as ~2 min for data capture and ~30 s for processing and conversion from gap data to band data. The camera sensor’ maximum frame rate, reported by the manufacturer, is 30 fps.

**Table A2:** Exact exposure time and WD settings during the study of each parameter.

Parameters	Exposure Time (ms)				WD (mm)			
	C-1	E-1	C-2	E-2	C-1	E-1	C-2	E-2
Ambient light	375	500	85	100	150	10	88	10
Exposure time	125, 250, 375, 500	125, 250, 375, 500	25, 50, 75, 100	50, 100, 150,200	150	10	100	10
Camera warm-up time	600	-	85	-	150	-	87	-
Spatial and temporal averaging	-	500	-	200	-	10	-	10
Camera focus	375	500	85	200	150	10	88	10
WD	375	500	70	200	150 ± 15	10 ± 4	90 ± 15	10 ± 4
$\theta_{ill}$	500	-	200	-	140	-	92	-
$\theta_{tar}$	350	500	85	100	161	15	85	13
